# Effects of Selenium Nanoparticles on Preventing Patulin-Induced Liver, Kidney and Gastrointestinal Damage

**DOI:** 10.3390/foods11050749

**Published:** 2022-03-04

**Authors:** Yue Qiu, Xinlu Chen, Zhangxi Chen, Xuejun Zeng, Tianli Yue, Yahong Yuan

**Affiliations:** 1 College of Food Science and Technology, Zhejiang University of Technology, Hangzhou 310014, China; qiuy@nwafu.edu.cn (Y.Q.); cxlzjut@126.com (X.C.); thegr8zx@163.com (Z.C.); 2 College of Food Science and Engineering, Northwest A&F University, Xianyang 712100, China; zxj1990@nwafu.edu.cn (X.Z.); ytl6503@163.com (T.Y.); 3 National Engineering Research Center of Agriculture Integration Test (Yangling), Xianyang 712100, China; 4 College of Food Science and Engineering, Northwest University, Xi’an 710069, China

**Keywords:** patulin, selenium nanoparticles, antioxidative, nephrotoxicity, hepatotoxicity, gastrointestinal damage

## Abstract

Patulin (PAT) is a toxic fungal metabolite, and oxidative damage was proved to be its important toxicity mechanism. Selenium nanoparticles (SeNPs) were prepared by reducing sodium selenite with chitosan as a stabilizer and used for preventing PAT-induced liver, kidney and gastrointestinal damage. SeNPs have good dispersibility, in vitro antioxidant activity, and are much less cytotoxic than sodium selenite. Cell culture studies indicated that SeNPs can effectively alleviate PAT-induced excessive production of intracellular ROS, the decline of glutathione peroxidase activity, and the suppression of cell viability. Evaluation of serum biochemical parameters, histopathology, oxidative stress biomarkers and activities of antioxidant enzymes in a mouse model showed that pre-treatment with SeNPs (2 mg Se/kg body weight) could ameliorate PAT-induced oxidative damage to the liver and kidneys of mice, but PAT-induced gastrointestinal oxidative damage and barrier dysfunction were not recovered by SeNPs, possibly because the toxin doses suffered by the gastrointestinal as the first exposed tissues exceeded the regulatory capacity of SeNPs. These results suggested that a combination of other strategies may be required to completely block PAT toxicity.

## 1. Introduction

Patulin (PAT) is an unsaturated heterocyclic lactone produced by several fungal species of *Penicillium*, *Aspergillus* and *Byssochlamys*. This water-soluble and thermostable mycotoxin is present in many vegetables and fruits (especially apples), as well as in cereals, tea, and silage [1]. Although PAT was originally discovered as a wide-spectrum antibiotic, it was reclassified as a hazard for its potential negative health effects. Acute, chronic, and cellular toxicities of PAT have been reported by a wide number of studies [2]. The acute symptoms of PAT consumption include agitation, convulsions, vomiting, and organ damage such as kidney, liver, lung and gastrointestinal [3]. Chronic health risks of PAT exposure involve neurotoxicity, immunotoxicity, genotoxicity, teratogenicity, and carcinogenicity. The toxic effects of PAT at cellular level include causing DNA damage and protein inactivation, inhibiting the synthesis of some key enzymes, and inducing apoptosis, etc. [4]. 

The details of the toxic effects of PAT remain controversial, as studies have not been standardized. Different cell and animal models, as well as the implementation of different dosages and administration routes (oral, intraperitoneal or subcutaneous injection) often lead to a lack of comparability of results [5]. Most of these studies, nevertheless, reveal that inducing oxidation stress is a vital toxic mechanism [6]. In order to alleviate the adverse health effects of PAT, antioxidants such as ascorbic acid [7], vitamin E [8], 6-gingerol [9], tea polyphenols [10], apigenin [11] and crocin [12] have been recommended for treatment. Moreover, as PAT exposure causes glutathione (GSH) depletion, GSH supplementation [13] has also been used to treat PAT toxicity.

Selenium (Se) is an essential trace mineral for health and a well-known antioxidant [14]. Se exists in organic (e.g., selenomethionine (SeMet), selenocysteine and methylseleninic acid (MSeA)), inorganic (e.g., selenite and selenate) and elemental forms, all of which vary in toxicity and bioavailability [15]. Selenium supplements have been shown to alleviate the damage induced by several mycotoxins (including PAT) through its antioxidant activity [16]. There are two reports on the protective effects of sodium selenite (Na_2_SeO_3_), SeMet and MSeA on PAT-induced organic oxidative damage in mice [17,18], but the studies did not examine the gastrointestinal toxicity of PAT. Moreover, these two studies administrated PAT to the subject by intraperitoneal injection, which is different from a natural exposure method.

The toxicity of selenium supplements remains a great concern due to the narrow range between its toxicity and effective concentrations [19]. Studies have revealed that organic selenium generally has lower toxicity and higher bioavailability than inorganic selenium [20]. Selenium nanoparticles (SeNPs) is a new form of selenium supplement, which encapsulates zero-valent selenium in suitable nano-vehicles (e.g., protein and polysaccharide) to maintain its stability [21]. Recent studies have shown that SeNPs have similar absorption and metabolism properties to organic selenium, and also have the advantages of low toxicity and high bioavailability [22,23]. It is worth noting that the cost of SeNPs is much lower than that of organic selenium, which provides new ideas for the field of selenium dietary supplements. However, there are no reports about the toxic antagonism effect of SeNPs against PAT so far.

Herein, SeNPs stabilized with chitosan were prepared using ascorbic acid as a reducing agent. The particle size, zeta potential and in vitro antioxidant properties of the as-prepared SeNPs were characterized. Subsequently, the preventive effects of SeNPs on PAT-mediated toxicity were evaluated, both in cells and animal models. In this study, the antioxidative activity of SeNPs on the mitigation of PAT-induced liver and kidney oxidative damage was studied, and the role of SeNPs on gastrointestinal injury induced by PAT was also concerned.

## 2. Materials and Methods

### 2.1. Chemicals and Reagents

Patulin was purchased from Sigma-Aldrich (St. Louis, MO, USA). Chitosan (CTS), 2,2′-Azino-bis (3-ethylbenzothiazoline-6-sulfonic acid (ABTS) and 2,2-Diphenyl-1-picrylhydrazyl (DPPH) were obtained from Aladdin Biochemical Technology Co., Ltd. (Beijing, China). Sodium selenite, ascorbic acid (Vitamin C, VC) and chloral hydrate were obtained from Sinopharm Chemical Reagent Co., Ltd. (Beijing, China). Dulbecco’s modified Eagle’s medium and penicillin-streptomycin solution were purchased from BasalMedia Technologies (Shanghai, China). Fetal bovine serum was obtained from Gemini Bio-Products (West Sacramento, CA, USA). A Cell Counting Kit-8 (CCK 8) was purchased from Abmole Bioscience (Houston, TX, USA). A reactive oxygen species (ROS) assay kit was obtainedfrom Beyotime Biotechnology (Shanghai, China). An ELISA Kit for lipopolysaccharides (LPS) were purchased from Xinlebio (Shanghai, China). Malondialdehyde (MDA), protein carbonyls (PC), and activities of antioxidative enzymes detection kits were purchased from Nanjing Jiancheng Bioengineering Institute (Nanjing, China).

### 2.2. SeNPs Preparation and Characterization

The preparation of SeNPs was according to a previous study with minor modifications [24]. CTS was dissolved into an acetic acid aqueous solution (1%, *w/w*) to obtain a CTS solution with a concentration of 1.0 mg/mL. Sodium selenite (20 mM) and VC (60 mM) were dissolved in the above CTS solution, respectively. Then, an equal volume of Na_2_SeO_3_ solution and the VC solution were mixed together to initiate a reaction and were stirred for 30 min to obtain a red SeNPs solution. As the theoretical molar ratio of the reaction is 1:2 between Na_2_SeO_3_ and VC, the actual reaction was mixed at a 1:3 ratio in order to avoid residual selenite in the system. A Se-free solution was prepared in the same way as SeNPs solution, except that no sodium selenite was added. The Se-free solution was used as a control to verify the physiological activity of selenium. All dosage descriptions for selenium supplements in this study were based on elemental selenium unless otherwise specified. The size, polydispersity index and zeta potential of the as-prepared SeNPs were measured by a laser granularity analyzer (Malvern ZEN3600, Worcestershire, UK).

### 2.3. In Vitro Antioxidant Activity

The antioxidant capacity of the SeNPs was estimated by DPPH and ABTS radical scavenging assays and ferric reducing antioxidant potential (FRAP) assay [25]. ABTS and DPPH radical scavenging activities were calculated using the following formula:

RS (%) = (A_b_ − A_s_)/A_b_ (1)

where A_s_ and A_b_ are absorbance of the samples with and without SeNPs, respectively.

The reducing capacity of SeNPs were measured by FRAP assay and calculated in relation to the absorbance signal from a FeSO_4_ solution. The FRAP value is expressed as the equivalent antioxidant capacity of 1.0 mM Fe^2+^.

### 2.4. Cell Culture and Treatments

Human hepatocyte LO2 cells and human embryonic kidney 293 cells (HEK 293) were cultured in Dulbecco’s modified Eagle’s medium supplemented with 10% fetal bovine serum and 1% penicillin-streptomycin. When cells reached ~50% confluence, PAT and/or selenium supplements were applied. Selenium was administered 12 h before with PAT. Cell viability was measured using a Cell Counting Kit-8. Intracellular ROS levels and glutathione peroxidase (GPx) activities were determined using the commercial kits mentioned in Section 2.1.

### 2.5. Animal Experiments

#### 2.5.1. Animals and Treatments

Male C57BL/6 mice (6–8 weeks old) were purchased from the Experimental Animal Center, Xi’an Jiao Tong University (Xi’an, China). Animal care and procedures were approved by the Institution Animal Care and Use Committee of Northwest A&F University. All animals were fed a commercial standard cube diet and water ad libitum. The mice were randomly divided into 4 groups (*n* = 10 per group) after a week of acclimatization: (I) vehicle control (saline solution), (II) PAT, (III) SeNPs, and (IV) SeNPs and PAT.

SeNPs (2 mg Se/kg body weight/d) was administered by oral gavage for 5 d before PAT treatment. PAT was dissolved in saline solution and given by gastric intubation 3 h after the last SeNPs administration as a single dose of 30 mg/kg body weight. The mice were sacrificed 24 h after PAT treatment, after which blood samples were collected and centrifuged to obtain serum. Liver, kidney and gastrointestinal tissue were excised and washed in ice-cold saline. The tissues were then immediately frozen in liquid nitrogen and stored at −80 °C or fixed in 10% formalin. The formalin-fixed tissues were embedded in paraffin wax, sectioned at 5 to 7 μm thickness and stained with hematoxylin and eosin (H&E) for histopathological observation.

#### 2.5.2. Biochemical Parameters Determination

The activities of alanine aminotransferase (ALT) and aspartate aminotransferase (AST), and the levels of urea nitrogen (BUN), and uric acid (UA) in serum, were determined by a Hitachi 7180 automatic serum biochemical analyzer. LPS levels in the serum were measured using an ELISA kit.

The frozen liver and kidney tissues were homogenized according to commercial kits. The samples were centrifuged, and the resulting supernatants were used to detect the concentrations of GSH, glutathione disulfide (GSSG), MDA, PC, and antioxidant enzyme activities, including superoxide dismutase (SOD), catalase (CAT), glutathione reductase (GR), GPx, and glutathione S-transferase (GST).

### 2.6. Statistical Analysis

All data are presented as mean ± SD. Statistical significance was analyzed via one-way ANOVA followed by Duncan’s multiple range test using SPSS 23.0 software (SPSS Inc., Chicago, IL, USA); *p* < 0.05 was considered statistically significant.

## 3. Results

### 3.1. Characterization of SeNPs

**Figure 1:**
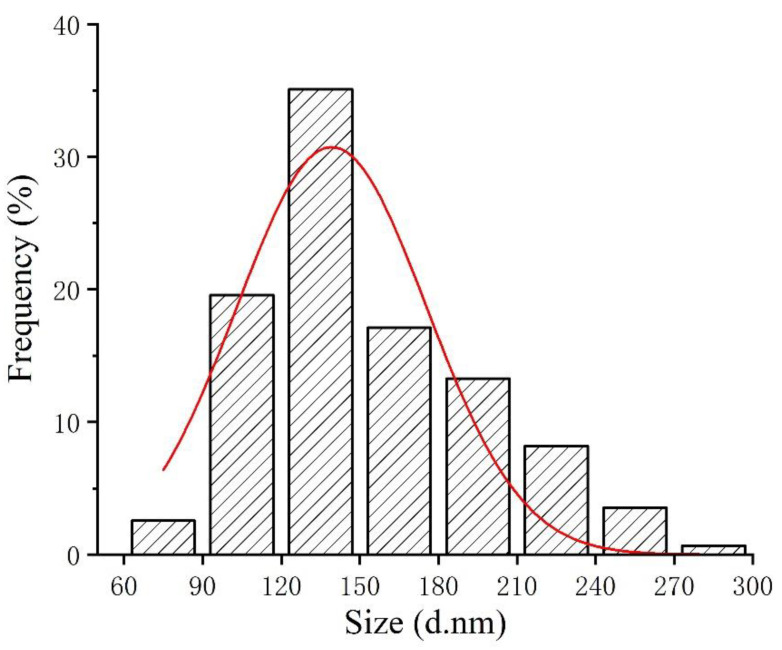
Particle size distribution map of SeNPs.

Studies indicated that the bioactivity of SeNPs was size dependent [26,27]. Therefore, particle size of the red SeNPs prepared in this study was examined by dynamic light scattering before application, revealing an average particle size of 133.4 ± 2.9 nm. The size distribution of SeNPs is shown in Figure 1. The polydispersity index value is 0.115 ± 0.016, indicating that the SeNPs were well-dispersed in solution. CTS with good biocompatibility was used as a stabilizer because element Se is unstable and easily converted to a black or gray inactive form [28]. The examination of zeta potential indicated that SeNPs was positively charged (40.5 ± 2.34 mV), due to the cationic property of CTS [24].

### 3.2. In Vitro SeNPs Antioxidant Activities

**Figure 2:**
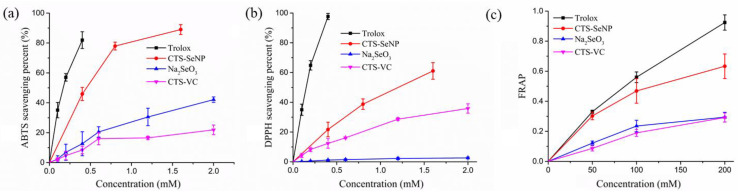
Antioxidant activity of SeNPs as determined by (**a**) ABTS, (**b**) DPPH and (**c**) FRAP assays. Trolox, Na_2_SeO_3_ and Se-free solution were control groups. The results are expressed as means ± SD.

In order to verify the physiological activity of selenium, Trolox and Se-free solution served as controls to exclude the possible influence of residual VC, VC oxidation products and CTS in the SeNPs preparation system. The antioxidant activities of Na_2_SeO_3_ were also evaluated, as it is the selenium source for SeNPs preparation. It can be seen that SeNPs had greater free radical scavenging capacity (ABTS, DPPH) and ferric reducing power in comparison to Na_2_SeO_3_ and Se-free solution (Figure 2). In addition, the SeNPs was better able to scavenge ABTS free radicals than DPPH at the same concentration.

### 3.3. Effect of SeNPs on PAT-Induced Cytotoxicity

#### 3.3.1. Cytotoxicity of Prepared SeNPs

**Figure 3:**
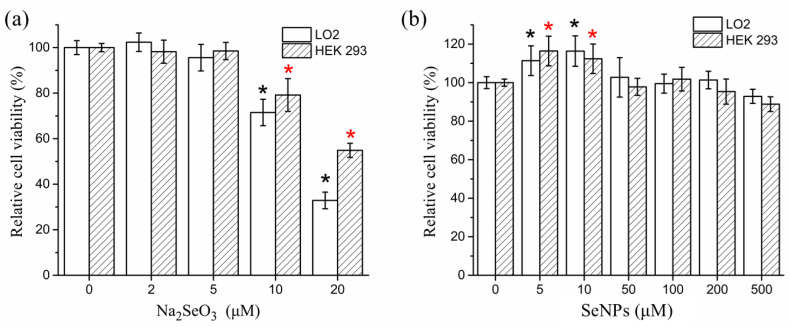
Cytotoxicity of (**a**) Na_2_SeO_3_ and (**b**) SeNPs in LO2 cells and HEK 293 cells. The results are expressed as means ± SD. * *p* < 0.05 versus control.

The dose-dependent effects of SeNPs and Na_2_SeO_3_ on cell viability of LO2 and HEK 293 are shown in Figure 3. No significant growth inhibition was observed at <500 μM SeNPs. In contrast, Na_2_SeO_3_ was much more toxic and significantly suppressed cell proliferation at 10 μM.

#### 3.3.2. Effect of SeNPs on PAT-Mediated Cytotoxicity

**Figure 4:**
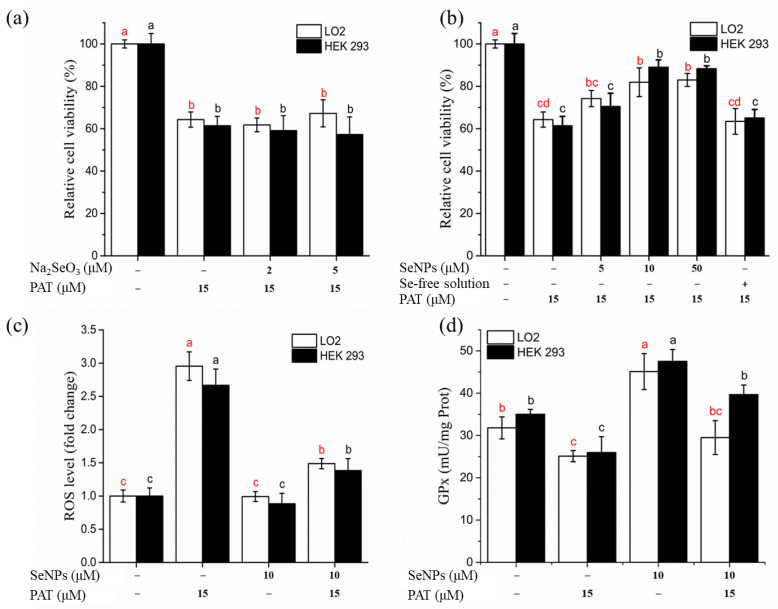
Effects of SeNPs on PAT-induced cytotoxicity in LO2 cells and HEK 293 cells. (**a**) Effect of Na_2_SeO_3_ on PAT-induced growth inhibitory of LO2 cells and HEK 293 cells. (**b**) Effect of SeNPs on PAT-induced growth inhibitory of LO2 cells and HEK 293 cells. (**c**) Effect of SeNPs on PAT-induced overproduction of ROS. (**d**) Effect of SeNPs on PAT-induced inhibition of intracellular GPx activity. The results are expressed as means ± SD. The different lowercase letters in the same color indicate significance (*p* < 0.05) of values.

Na_2_SeO_3_ (2 and 5 μM) and SeNPs (5, 10, and 50 μM) were used to investigate the protective effect of selenium supplementation on PAT cytotoxicity (Figure 4a,b) with the concentrations that were determined to be safe in Section 3.3.1. PAT treatment alone significantly (*p* < 0.05) decreased cell viability for both LO2 and HEK 293 cells. Cells treated with 10 and 50 μM SeNPs 12 h before PAT administration significantly (*p* < 0.05) increased the percentage of viable cells compared with PAT treatment alone. In contrast, cells pre-treated with Na_2_SeO_3_ were not significantly (*p* > 0.05) different from the PAT control cells. Pre-treatment cells with Se-free solution also had no obvious alleviated effect on PAT-induced cytotoxicity, suggesting the pivotal role of selenium. Based on these results, cells were pre-treated with 10 μM SeNPs for all subsequent experiments.

ROS overproduction has been linked to PAT-caused apoptosis [29]. As shown in Figure 4c, exposure to PAT induced a significant increase (approximately 3-fold) in intracellular ROS levels, while SeNPs pretreatment reduced the PAT-induced ROS overproduction. Incubation with SeNPs alone did not cause excessive ROS generation.

Figure 4d shows intracellular GPx activity with different treatments. PAT administration caused a significant inhibition of GPx (an antioxidant selenoenzyme) activity, while SeNPs treatment increased GPx activity. Pre-treatment with SeNPs attenuated the PAT-mediated decrease in GPx activity.

### 3.4. Effects of SeNPs on Liver and Kidney Injury Induced by PAT in Mouse Model

After preliminary validation of the mitigating effect of SeNPs on PAT toxicity by the cellular model, the protective effects of SeNPs against PAT toxicity were further investigated in the animal model.

#### 3.4.1. Serum Biochemistry Parameters

ALT and AST are commonly used as indicators of liver disease, including PAT induced liver injury [30]. PAT administration significantly (*p* < 0.05) elevated ALT and AST levels, while SeNPs could restore these indicators to normal (Table 1). On the contrary, BUN and UA, as kidney functional parameters [31], were not affected by PAT. Treatment with SeNPs alone had no significant (*p* > 0.05) effect on the four serum biochemical parameters compared with the control group.

#### 3.4.2. Histopathology of Liver and Kidney

**Figure 5:**
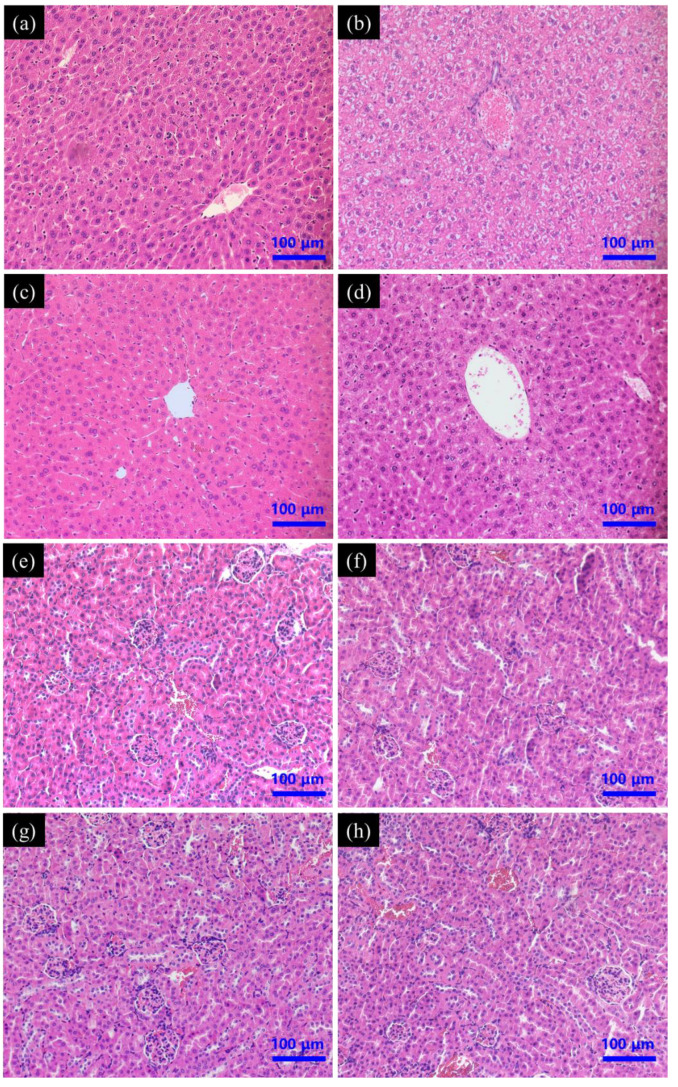
Effect of SeNPs on PAT-evoked pathological damage of (**a**–**d**) liver and (**e**–**h**) kidney tissue as assessed by H&E staining. Control group (**a**,**e**); PAT treatment group (**b**,**f**); SeNPs treatment group (**c**,**g**); SeNPs pre-treatment followed by PAT administration (**d**,**h**).

The liver of mice from the control group had no noticeable histological changes; the structure of the hepatic lobule was clear and the hepatic cord around the central vein was radially arranged. Hepatocytes were polygonal with round nuclei and clear nuclear membranes (Figure 5a). Liver from PAT treatment group displayed abnormal hepatocyte morphology and extensive vacuolar degeneration, with prevalent fragmentation, hepatocyte dissolution, and cell nuclei lysis (Figure 5b). Treatment with only SeNPs did not cause overt changes in the liver histology (Figure 5c). Pre-treatment with SeNPs prior to PAT administration greatly reduced the liver lesions caused by PAT, although some cells had mild vacuolation and nuclear lysis, principally distributed near the central vein (Figure 5d). No kidney histological changes were observed in either the control group or treated groups (Figure 5e–h).

#### 3.4.3. Oxidative Stress Biomarkers of Liver and Kidney

**Figure 6:**
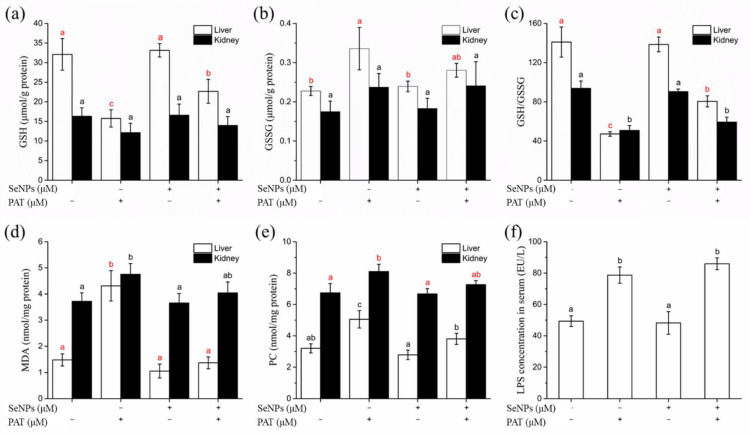
The effect of SeNPs on oxidative stress indicators in liver and kidney of mice, including (**a**) GSH, (**b**) GSSG, (**c**) GSH/GSSG, (**d**) MDA and (**e**) PC levels, as well as (**f**) serum LPS levels. The results are expressed as means ± SD. The different lowercase letters in the same color indicate significance (*p* < 0.05) of values.

MDA is one of the final products of unsaturated fatty acids peroxidation [32]. PAT significantly (*p* < 0.05) increased MDA levels in the liver and kidney, especially in hepatic tissue, where MDA concentration increased ~3 fold that of the control group (Figure 6d). SeNPs pre-treatment before PAT administration restored MDA to normal levels in both liver and kidney, demonstrating that SeNPs treatment effectively decreased PAT-induced lipid peroxidation.

Protein oxidation in tissue usually results in increased levels of protein carbonyls, a relatively stable marker of tissue oxidative damage [33]. PAT administration caused significant (*p* < 0.05) increases in PC levels in hepatic and renal tissues, while SeNPs pre-treatment prevented the PAT-mediated oxidation of protein in tissues (Figure 6e).

GSH is a small endogenous thiol-containing molecule, and the GSH system is of great importance for maintaining redox homeostasis [34]. PAT toxicity has been reported to be related to inducing depletion of GSH [13]. PAT administration significantly (*p* < 0.05) reduced GSH level in hepatic tissue by a half compared to the control group, while GSH level in kidney decreased but did not reach a significant (*p* > 0.05) level (Figure 6a). Additionally, the GSSG level in the liver tissue of the PAT-treated group was remarkably increased, but not in the kidney (Figure 6b). Glutathione redox status (GSH/GSSG) decreased both in the liver and kidney after PAT administration (Figure 6c). SeNPs pre-treatment attenuated the PAT-induced disorder of glutathione homeostasis, but did not return GSH or the GSH/GSSG ratio back to normal levels.

GPx, GR, and GST are antioxidant enzymes that are essential to glutathione metabolism. GPx, an important selenoenzyme, can scavenge a various of peroxides [35], while GR utilizes NADPH to catalyze the reduction of GSSG to GSH [36]. GST plays a detoxifying role by catalyzing the binding reaction of glutathione to various foreign chemicals [37], and its interaction network may be a pharmacological target of selenium compounds [38]. Oral administration of PAT caused a significant (*p* < 0.05) decrease in the activity of all three antioxidant enzymes in the liver (Table 2), while only GPx activity in kidney tissue was significantly (*p* < 0.05) lower than the control group (Table 3). Pre-treatment with SeNPs effectively reversed the diminished GPx and GST activity in the liver, in addition to restoring GPx activity in renal tissues. However, SeNPs had a barely noticeable impact on GR activity.

SOD and CAT are two other important antioxidant enzymes. Exposure to PAT significantly (*p* < 0.05) reduced SOD and CAT activities in the liver (Table 2), while the activities of these two enzymes did not change obviously in the kidney (Table 3). SeNPs reversed the PAT-induced the decrease in SOD activity in hepatic tissue back to control levels, but did not markedly improve CAT activity.

### 3.5. Effect of SeNPs on PAT-Induced Gastrointestinal Damage

#### 3.5.1. Histopathology of Gastrointestinal Tract

**Figure 7:**
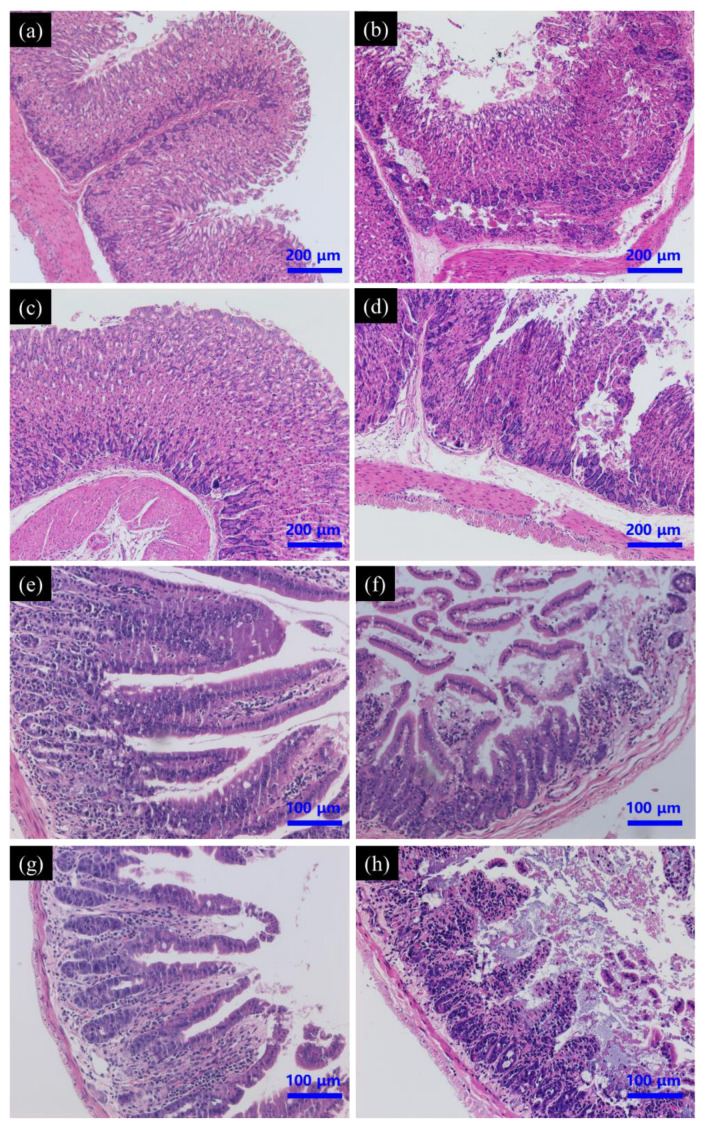
Effect of SeNPs on PAT-evoked pathological damage of stomach (**a**–**d**) and intestine (**e**–**h**) as assessed by H&E staining. Control group (**a**,**e**); PAT treatment group (**b**,**f**); SeNPs treatment group (**c**,**g**); SeNPs pre-treatment followed with PAT administration (**d**,**h**).

Gastrointestinal pathology was evaluated in all groups to determine whether SeNPs could reverse or reduce PAT-induced gastric mucosal damage. Histologically, PAT treatment caused extensive damage to the gastric mucosa, while gastric sections demonstrated epithelial cell necrosis, submucosal edema and infiltration with inflammatory cells. Pre-treatment with SeNPs had no effect on PAT-induced gastric mucosal injury. PAT also caused intestinal injury, including inflammatory cell infiltration, desquamation, and degeneration of intestinal villi and edema in submucosa. These pathological changes were also not restored by SeNPs supplementation (Figure 7).

#### 3.5.2. LPS Level

The serum endotoxin (lipopolysaccharide, LPS) level was measured since it is considered to be an indicator of gut barrier dysfunction [39]. PAT administration dramatically increased the serum LPS levels (Figure 6f), indicating damage to the intestinal barrier. However, pre-treatment with SeNPs did not reduce serum LPS to a normal level, suggesting that SeNPs would not protect mice from PAT-induced endotoxemia.

#### 3.5.3. Oxidative Stress Biomarkers of Gastrointestinal Tissues

MDA and PC level and the activities of two antioxidant enzymes in the gastrointestinal were further determined (Table 4). Exposure to PAT significantly changed the levels of these biochemical parameters. However, except for PC levels in intestinal, all the oxidative stress biomarkers tested could not been recovered to normal levels by SeNPs administration.

## 4. Discussion

The objective of this study was to investigate the potential protective effects of SeNPs on PAT toxicity, both in cell culture and animal models. We included PAT-induced gastrointestinal injury in the evaluation, instead of focusing only on preventing oxidative stress. Pre-treatment with SeNPs reduced liver and kidney oxidative damage caused by PAT administration, but did not diminish or reverse PAT-induced gastrointestinal lesions.

The SeNPs used in this work was prepared by ascorbic acid reduction with sodium selenite as the selenium source and CTS as the stabilizer. Red element selenium is prone to aggregate into black or gray form and loses biological activity [39], which can be controlled by some macromolecules with good biocompatibility, such as proteins, polysaccharides and polyphenols [21]. SeNPs prepared with polysaccharide can be administered orally, while SeNPs stabilized by protein or polyphenol are usually unstable and cannot resist low pH and enzymolysis [40]. CTS is the only positively charged natural alkaline polysaccharide [41] which can increase SeNP absorption through electrostatic interactions with negatively charged intestinal mucin and cell membranes [42].

SeNPs displayed protective effects against PAT-induced oxidative stress, both in cell culture and animal models. The antioxidant efficacy of selenium was believed to be mainly attributed to the regulation of antioxidant enzymes, especially selenium-containing enzymes [43,44]. This study revealed that SeNPs treatment rescued some of the activity of certain antioxidant enzymes (especially GPx) that had been inhibited by PAT, which was consistent with the study reported by Song et al., that selenium significantly diminished the inhibition of PAT-caused GPx mRNA expression levels [18]. Interestingly, Song et al. indicated that Na_2_SeO_3_ protected the brains of mice against PAT-induced oxidative damage [18], whereas both the report by Lu et al. [17] and the present study showed that 5 μM inorganic selenium cannot antagonize PAT-induced cytotoxicity. Lu et al. presumed that the deficient selenium status of the mice used in the study of Song et al., led to the difference [17]. However, we compared the details in different studies and found that both Lu et al., and us the current study used Na_2_SeO_3_ only in cell culture and eliminated this inorganic selenium supplementation in subsequent animal models because the expected effect was not obtained. According to the results of some recent selenium metabolism studies, the organic selenium recommended by Lu et al. and the nano-selenium used by us has the same metabolism way that can be directly absorbed, while the absorption of inorganic selenium used by Song et al. relied on microbial transformation [22,23]. Therefore, we speculated that this is the main reason for the inability of Na_2_SeO_3_ to reverse PAT-induced cytotoxicity in cell culture. In addition, using 5 µM SeNPs (the maximum nontoxic dose of Na_2_SeO_3_) also failed to protect cells from PAT-induced suppression of cell proliferation. It could be surmised that the inability of Na_2_SeO_3_ to protect against the cytotoxicity of PAT may be due to inadequate dosing, although it was the last resort considering its toxicity range in the cellular system. These conflicting results suggest that the metabolic pathway and absorption efficiency of different selenium forms and the suitability of the selected experimental model should be fully considered when evaluating the activity of selenium.

The digestive tract is the first set of tissues that are exposed to PAT orally, which means that gastrointestinal tissues would encounter high concentrations of the toxin. In some toxicological studies (experiments with mice or rats), gastrointestinal ulcer and inflammation were the only pathological changes found in PAT exposure [45,46], and some researchers speculated that PAT toxicity should be partly attributed to enterotoxaemia [47,48]. The results of this study indicated that PAT caused gastrointestinal pathological damage and elevated serum LPS levels, indicating gut barrier dysfunction, which was consistent with two recent studies [48,49]. However, SeNPs had little effect on PAT-induced gastrointestinal damage. Since there was no data to confirm whether PAT-induced oxidative stress was directly related to gastrointestinal damage, it cannot be determined whether the failure of SeNPs to alleviate the gastrointestinal injury is because PAT-induced oxidative stress is not the main factor leading to gastrointestinal injury. Therefore, some oxidative stress biomarkers in the gastrointestinal tract of mice in different treatment groups were further determined. The results indicated that PAT indeed caused gastrointestinal oxidative damage, while SeNPs was unable to relieve the changes in these parameters like in liver tissues. This suggested that the inability of SeNPs to PAT-induced gastrointestinal damage might be due to the fact that the toxin dose that the digestive tract was exposed to exceeded the regulatory capacity of SeNPs. In addition, CTS might promote absorption of PAT through the digestive tract, as it possesses mucoadhesive properties [41] and can induce short-term opening of tight junction proteins [50,51], which may be an unintended side effect of increasing selenium absorption efficiency. It could be conjected that replacing the stabilizer used to prepare SeNP and utilizing SeNP, together with gastrointestinal protective substances, may obtain better treatment efficacy.

In conclusion, pre-treatment with SeNPs ameliorated many of the negative impacts of PAT in cell culture and animal models, while it should be noticed that this protective effect did not occur in gastrointestinal tissues, which indicated that complete prevention of PAT toxicity will require an integrated approach. It also suggested the necessity to focus on gastrointestinal toxicity of PAT in future studies. Moreover, an in-depth understanding of the mechanism of PAT toxicity is also necessary to develop more effective prevention or therapeutic strategies.

## Figures and Tables

**Table 1:** Serum biochemical parameters of the control and trial groups *.

	ALT (U/L)	AST (U/L)	BUN (mmol/L)	UA (mmol/L)
Control	26.47 ± 2.98 a	79.07 ± 7.39 a	10.54 ± 1.05 a	88.33 ± 10.62 a
PAT	43.70 ± 4.64 b	98.50 ± 8.00 b	11.75 ± 0.77 a	88.33 ± 15.63 a
SeNPs	26.33 ± 1.30 a	80.13 ± 5.73 a	10.75 ± 0.88 a	95.67 ± 10.66 a
SeNPs + PAT	29.33 ± 3.91 a	87.70 ± 3.36 ab	11.22 ± 1.25 a	94.00 ± 3.74 a

* The results are expressed as means ± SD. The different lowercase letters in the same colum indicate significance (*p* < 0.05) of values.

**Table 2:** Effect of SeNPs on SOD, CAT, GPx, GR and GST activities in liver of PAT-treated mice *.

	SOD (U/mg Prot)	CAT (U/mg Prot)	GPx (U/mg Prot)	GR (U/mg Prot)	GST (U/mg Prot)
Control	310.07 ± 31.07 a	27.76 ± 2.49 a	2.61 ± 0.31 b	39.82 ± 3.99 a	6.27 ± 0.53 b
PAT	220.05 ± 25.00 b	21.50 ± 1.75 b	0.80 ± 0.19 c	27.79 ± 3.48 b	3.76 ± 0.49 c
SeNPs	306.69 ± 15.62 a	26.08 ± 2.73 ab	3.37 ± 0.29 a	38.04 ± 3.27 a	8.57 ± 0.67 a
SeNPs + PAT	288.75 ± 23.43 a	22.17 ± 0.74 b	2.88 ± 0.36 ab	27.60 ± 1.28 b	5.37 ± 0.61 b

* The results are expressed as means ± SD. The different lowercase letters in the same colum indicate significance (*p* < 0.05) of values.

**Table 3:** Effect of SeNPs on SOD, CAT, GPx, GR and GST activities in kidney of PAT-treated mice *.

	SOD (U/mg Prot)	CAT (U/mg Prot)	GPx (U/mg Prot)	GR (U/mg Prot)	GST (U/mg Prot)
Control	217.00 ± 32.34 a	16.88 ± 1.31 a	2.13 ± 0.23 b	38.57 ± 1.36 a	4.09 ± 0.53 a
PAT	177.90 ± 17.61 a	17.44 ± 3.01 a	1.48 ± 0.16 c	36.61 ± 3.57 a	3.82 ± 0.75 a
SeNPs	169.80 ± 30.54 a	16.30 ± 0.67 a	2.64 ± 0.12 a	36.10 ± 6.28 a	5.14 ± 0.76 a
SeNPs + PAT	190.58 ± 19.74 a	15.78 ± 2.57 a	2.18 ± 0.17 b	33.30 ± 2.71 a	4.59 ± 0.86 a

* The results are expressed as means ± SD. The different lowercase letters in the same colum indicate significance (*p* < 0.05) of values.

**Table 4:** Effect of SeNPs on MDA and PC levels and the activities of SOD and GPx in gastric and intestinal of PAT-treated mice *.

	MDA (U/mg Prot)	GPx (U/mg Prot)	GR (U/mg Prot)	GST (U/mg Prot)
**Gastric**				
Control	1.75 ± 0.39 a	2.31 ± 0.42 a	200.41 ± 11.10 a	1.94 ± 0.16 a
PAT	4.49 ± 0.80 b	4.33 ± 1.09 b	160.80 ± 12.03 b	1.57 ± 0.04 b
SeNPs	1.52 ± 0.18 a	2.26 ± 0.23 a	220.85 ± 21.18 a	1.94 ± 0.14 a
SeNPs + PAT	3.96 ± 0.45 b	4.32 ± 0.65 b	153.18 ± 6.53 b	1.64 ± 0.12 b
**Intestinal**				
Control	1.29 ± 0.23 a	2.83 ± 0.75 ab	189.88 ± 21.00 a	1.54 ± 0.25 a
PAT	4.62 ± 0.84 c	5.00 ± 1.12 c	139.75 ± 15.69 b	0.82 ± 0.19 b
SeNPs	1.00 ± 0.38 a	1.99 ± 0.59 a	192.76 ± 16.80 a	1.97 ± 0.22 a
SeNPs + PAT	3.20 ± 0.67 b	4.03 ± 0.78 bc	143.26 ± 13.38 b	1.00 ± 0.20 b

* The results are expressed as means ± SD. The different lowercase letters in the same colum indicate significance (*p* < 0.05) of values.

## Data Availability

Not applicable.

## Abbreviations and Acronyms

BUN	urea nitrogen
CAT	catalase
CCK 8	Cell Counting Kit-8
CTS	chitosan
DPPH	2,2-Diphenyl-1-picrylhydrazyl
FRAP	ferric reducing antioxidant potential
GPx	glutathione peroxidase
GR	glutathione reductase
GSH	glutathione
GSSG	glutathione disulfide
GST	glutathione S-transferase
LPS	lipopolysaccharides
MDA	malondialdehyde
MSeA	methylseleninic acid
Na_2_SeO_3_	sodium selenite
NADPH	nicotinamide adenine dinucleotide phosphate
PAT	patulin
PC	protein carbonyls
ROS	reactive oxygen species
Se	selenium
SeMet	selenomethionine
SeNPs	selenium nanoparticles
SOD	dismutase
UA	uric acid
VC	Vitamin C/ascorbic acid
